# Twenty-eight-day in-hospital mortality prediction for elderly patients with ischemic stroke in the intensive care unit: Interpretable machine learning models

**DOI:** 10.3389/fpubh.2022.1086339

**Published:** 2023-01-12

**Authors:** Jian Huang, Wanlin Jin, Xiangjie Duan, Xiaozhu Liu, Tingting Shu, Li Fu, Jiewen Deng, Huaqiao Chen, Guojing Liu, Ying Jiang, Ziru Liu

**Affiliations:** ^1^Department of General Surgery, The Second Xiangya Hospital, Central South University, Changsha, China; ^2^Guangxi University of Chinese Medical, Nanning, China; ^3^Health Management Center, The Second Xiangya Hospital of Central South University, Changsha, China; ^4^Department of Infectious Diseases, The First People's Hospital of Changde City, Changde, China; ^5^Department of Cardiology, The Second Affiliated Hospital of Chongqing Medical University, Chongqing, China; ^6^Key Laboratory of Neurological Diseases, The Second Affiliated Hospital of Xuzhou Medical University, Xuzhou, China; ^7^Army Medical University (The Third Military Medical University), Chongqing, China; ^8^Key Laboratory of Novel Materials for Sensor of Zhejiang Province, College of Materials and Environmental Engineering, Hangzhou Dianzi University, Hangzhou, China; ^9^Department of Neurosurgery, Xiu Shan People's Hospital, Chongqing, China; ^10^Department of Neurosurgery, University-Town Hospital of Chongqing Medical University, Chongqing, China; ^11^Department of Neurology, Southwest Hospital, Third Military Medical University (Army Medical University), Chongqing, China

**Keywords:** ischemic stroke, elderly patients, machine learning, hospital mortality, prediction model

## Abstract

**Background:**

Risk stratification of elderly patients with ischemic stroke (IS) who are admitted to the intensive care unit (ICU) remains a challenging task. This study aims to establish and validate predictive models that are based on novel machine learning (ML) algorithms for 28-day in-hospital mortality in elderly patients with IS who were admitted to the ICU.

**Methods:**

Data of elderly patients with IS were extracted from the electronic intensive care unit (eICU) Collaborative Research Database (eICU-CRD) records of those elderly patients admitted between 2014 and 2015. All selected participants were randomly divided into two sets: a training set and a validation set in the ratio of 8:2. ML algorithms, such as Naïve Bayes (NB), eXtreme Gradient Boosting (xgboost), and logistic regression (LR), were applied for model construction utilizing 10-fold cross-validation. The performance of models was measured by the area under the receiver operating characteristic curve (AUC) analysis and accuracy. The present study uses interpretable ML methods to provide insight into the model's prediction and outcome using the SHapley Additive exPlanations (SHAP) method.

**Results:**

As regards the population demographics and clinical characteristics, the analysis in the present study included 1,236 elderly patients with IS in the ICU, of whom 164 (13.3%) died during hospitalization. As regards feature selection, a total of eight features were selected for model construction. In the training set, both the xgboost and NB models showed specificity values of 0.989 and 0.767, respectively. In the internal validation set, the xgboost model identified patients who died with an AUC value of 0.733 better than the LR model which identified patients who died with an AUC value of 0.627 or the NB model 0.672.

**Conclusion:**

The xgboost model shows the best predictive performance that predicts mortality in elderly patients with IS in the ICU. By making the ML model explainable, physicians would be able to understand better the reasoning behind the outcome.

## Introduction

Ischemic stroke (IS) approximately accounts for 80% of strokes in elderly patients, which has become the second most serious cause of death in the world (1). Patients may need intensive care unit (ICU) treatment due to stroke-associated cerebral damage, concomitantly compromising other vital organ functions. As treatment and support options expand, the need for intensive care and acute stroke care will increasingly intertwine, and the number of stroke patients admitted to the ICU will rise (2). Notably, short-term mortality rates for patients with stroke who require intensive care treatment were high, and the survival curve gradient stabilized over time. In ICU-admitted patients with stroke, the 30-day mortality rate was 31% for ischemic stroke (3). Consequently, it is imperative to determine the risk of early death in the course of treatment for patients with IS who w admitted to the ICU.

Some models have also been developed to predict in-hospital mortality in cases of acute ischemic stroke. Mittal and Goel (4) reported a predictive score by investigating 188 consecutive patients with IS, and their predictive factors included admission, hypoxia (saturation of oxygen < 94%), National Institute of Health Stroke Scale (NIHSS) score >15, modified Rankin score (mRS) >3, Glascow Coma Scale (GCS) < 8, hyperglycemia (random blood sugar (RBS) >200 mg/dL), raised total leukocyte count (TLC), and high-sensitivity C-reactive protein (HS-CRP) (>10 mg/L). Both Saposnik et al. (5) and O'Donnell et al. (6) developed good models for acute IS patients. Wang et al. (7) reported an xgboost model with 30 variables, better than the LR reference model 0.891.

However, these models were not applicable to the elderly population in the ICU, and the performance of the model predicting the mortality among the elderly admitted to the ICU was unknown. The elderly patients should be given more attention because the majority of ischemic stroke cases occur in these elderly patients, while young patients who suffer from IS have a low mortality rate. In addition, with prolonged life expectancy, the aging population experiences a significant increase in stroke incidence. Additionally, elderly patients exhibit physiological changes including neuronal plasticity and decreased repair ability, as well as changes in the structure of the vascular system and complications, which increase the complexity of prediction. Limited data regarding short-term mortality predictors for elderly patients with IS are available. A model based on the LR method was constructed with 469 older patients (8). Furthermore, based on the LR method, Tuttolomondo et al. (9) reported that age, white blood cell (WBC) count, glucose blood level at admission, and Charlson comorbidity index score were directly associated with in-hospital mortality in the elderly.

Several factors were found to be associated with the increased short-term mortality, including age (5), sex (5), stroke severity and subtype (5), smoking (5), atrial fibrillation (5), serum calcium (10), serum troponin (11), Chronic Kidney Disease (12–15), hypertension (15), the National Institute of Health Stroke Scale (NIHSS) (15, 16), neutrophil-to-lymphocyte ratio (17), low triiodothyronine (T3) (18), hyperglycemia (19, 20), plasma brain natriuretic peptide (21), non-alcoholic fatty liver disease (NAFLD) (22), and elevated blood urea nitrogen (BUN)-to-creatinine ratio (23). With numerous variables involved, the complexity of stroke data lends itself well to machine learning (ML) algorithms that incorporate these variables into a predictive model (24). ML algorithms are thought to outperform clinical prediction models based on regression because they make fewer assumptions and can learn complex relationships between predictors and outcomes (25).

In this study, we aimed to develop prediction models for 28-day in-hospital mortality in elderly patients with IS using ML algorithms. The model was based on variables collected at admission. This will improve clinical decision-making and healthcare quality through early risk stratification after acute IS in elderly patients.

## Methods

### Design and participants

Data of elderly patients with IS were extracted from the eICU Collaborative Research Database (eICU-CRD) version 2.0 (26). All data were extracted from the eICU Collaborative Research Database (eICU-CRD, https://eicu-crd.mit.edu/) (certification ID: 42039823). The eICU-CRD database is a publicly available multi-center critical care database made available by Philips Healthcare in partnership with the MIT Laboratory for Computational Physiology and contains de-identified clinical data of over 200,000 patients admitted in the ICU from 2014 to 2015. Individuals were selected if their hospital discharge records contained at least one of the following: International Classification of Diseases (ICD)-9-Clinical Modification (CM) (ICD-9-CM) diagnoses of IS: 434.91 (cerebral artery occlusion, unspecified with cerebral infarction) and IS: I63.50 (cerebral infarction due to unspecified occlusion or stenosis of unspecified cerebral artery). The inclusion criteria for the present study were as follows: (1) first-ever ICU admission and (2) age ≥ 65 years. The exclusion criteria for the present study were as follows: (1) ICU stay < 24 h or more than 28 days; (2) individuals with severe liver disease; (3) individuals with heart failure; (4) individuals with metastatic solid tumor; and (5) individuals with more than 30% of missing values. With the clinical information from patients de-identified, the database's official ethics committee has approved the public release of these clinical data. A consent waiver was also given because of anonymized retrospective patient data.

### Outcome variables and predictors

The primary outcome event was in-hospital death within 28 days in elderly ICU patients with IS. The clinical data were collected within 24 h of admission. To identify candidate predictor variables, a review of literature was done and the present study selected variables that were available in the eICU. The finally selected 51 variables, including vital signs, demographics, laboratory tests, and comorbidities, are listed in Table 1. To ensure the accuracy of the results, variables with more than 30% missing values are excluded, and the K-Nearest Neighbor algorithm (KNN) is used to fill in those missing values.

**Table 1:** The population demographics and clinical characteristics.

**Variables**	**Total** **(*n* = 1,236)**	**Survival** **(*n* = 1,072)**	**Death** **(*n* = 164)**	***P-*value**
Age, Median (Q1, Q3)	77 (71, 84)	77 (71, 84)	77 (71.75, 85)	0.544
**Gender**, ***N*** **(%)**				0.514
Women	636 (51)	556 (52)	80 (49)	
Men	600 (49)	516 (48)	84 (51)	
**Ethnicity**, ***N*** **(%)**				0.181
Non-white	256 (21)	229 (21)	27 (16)	
White	980 (79)	843 (79)	137 (84)	
BMI, Median (Q1, Q3)	26.74 (23.34, 31.14)	26.66 (23.31, 30.97)	27.25 (23.68, 31.55)	0.535
Aniongap, median (Q1, Q3)	10 (8, 13)	10 (8, 13)	10 (8, 13)	0.312
Albumin, median (Q1, Q3)	3.65 (3.35, 3.85)	3.7 (3.4, 3.85)	3.55 (3.2, 3.8)	< 0.001
Bilirubin, median (Q1, Q3)	0.55 (0.45, 0.7)	0.55 (0.45, 0.7)	0.6 (0.5, 0.8)	0.002
ALT, median (Q1, Q3)	21.75 (17, 27)	21.5 (17, 26.5)	23.25 (18.5, 29)	0.005
AST, median (Q1, Q3)	22 (18.5, 27)	21.5 (18, 26)	24.75 (20, 35.5)	< 0.001
Alp, median (Q1, Q3)	76 (69.5, 91.5)	75.5 (69.5, 91)	81 (71, 94)	0.014
BUN, median (Q1, Q3)	20 (15, 25)	19 (15, 25)	23 (19, 31)	< 0.001
Creatinine, median (Q1, Q3)	1 (0.81, 1.3)	1 (0.8, 1.27)	1.13 (0.84, 1.4)	< 0.001
Sodium, median (Q1, Q3)	139 (136, 141)	139 (136, 141)	139 (136, 141)	0.348
Calcium, median (Q1, Q3)	9 (8.5, 9.4)	9 (8.6, 9.4)	8.9 (8.4, 9.3)	0.039
Chloride, median (Q1, Q3)	104 (101, 107)	104 (101, 107)	104 (100, 106)	0.849
Potassium, median (Q1, Q3)	4 (3.7, 4.4)	4 (3.7, 4.4)	4.2 (3.8, 4.5)	0.006
Glucose, median (Q1, Q3)	126 (105, 155)	125 (104, 152)	134.5 (112, 194.25)	< 0.001
Bicarbonate, median (Q1, Q3)	25 (23, 27)	25 (23, 27)	24 (22, 27)	0.007
WBC, median (Q1, Q3)	8.8 (7.1, 11.6)	8.6 (7, 11.2)	10.92 (8.3, 13.9)	< 0.001
Hematocrit, median (Q1, Q3)	39.1 (35.3, 42.9)	39.1 (35.5, 42.92)	38.7 (34.75, 42.35)	0.485
Hemoglobin, median (Q1, Q3)	13 (11.7, 14.4)	13.1 (11.7, 14.4)	12.9 (11.6, 14.2)	0.504
Platelets, median (Q1, Q3)	211 (174, 260)	210 (176, 260)	212.5 (166.75, 260.5)	0.783
INR, median (Q1, Q3)	1.05 (1, 1.11)	1.04 (1, 1.1)	1.1 (1, 1.2)	< 0.001
PTT, median (Q1, Q3)	28 (26.4, 30)	28 (26.6, 30)	27.9 (25.58, 30.8)	0.416
RBC, median (Q1, Q3)	4.32 (3.88, 4.74)	4.33 (3.9, 4.75)	4.24 (3.8, 4.69)	0.196
Mcv, median (Q1, Q3)	91 (87.57, 94)	91 (87.4, 94)	91.05 (87.7, 95)	0.245
Mchc, median (Q1, Q3)	33.3 (32.5, 34)	33.3 (32.5, 34)	33.2 (32.5, 34.1)	1
Mch, median (Q1, Q3)	30.5 (29.1, 31.65)	30.5 (29, 31.6)	30.5 (29.3, 31.7)	0.538
Rdw, median (Q1, Q3)	13.93 (13.35, 14.8)	13.9 (13.3, 14.8)	14.1 (13.5, 14.9)	0.056
Lymphs.Pct, median (Q1, Q3)	19 (12, 25)	19.6 (13, 26)	15.35 (9, 20.89)	< 0.001
Monos.Pct, median (Q1, Q3)	7.57 (6.2, 9)	7.6 (6.5, 9)	7 (5.6, 8.7)	0.004
Eos, median (Q1, Q3)	1.45 (1, 2)	1.5 (1, 2.22)	1.1 (1, 1.8)	< 0.001
Polys, median (Q1, Q3)	69.4 (63.5, 77)	69 (63, 76.1)	72.53 (67.22, 83)	< 0.001
Total Protein, median (Q1, Q3)	6.7 (6.4, 7.15)	6.7 (6.4, 7.2)	6.75 (6.4, 7.06)	0.471
PT, median (Q1, Q3)	13 (11.9, 14)	12.95 (11.85, 13.8)	13.3 (12.44, 14.43)	< 0.001
Triglycerides, median (Q1, Q3)	104 (79, 129)	104 (78, 129)	101.25 (81, 130.25)	0.882
Total cholesterol, median (Q1, Q3)	156.25 (134, 178)	156 (135, 178)	159.25 (125, 180)	0.746
Heart rate, median (Q1, Q3)	78.25 (69, 92)	78 (68, 91)	84 (72, 99.25)	< 0.001
Respiratory rate, median (Q1, Q3)	18 (16, 21)	18 (16, 21)	18 (16, 22)	0.369
SpO_2_, median (Q1, Q3)	97 (96, 99)	97 (96, 99)	98 (96.5, 99)	0.012
Nibp.Systolic, median (Q1, Q3)	151 (131, 166.25)	151 (131, 166)	151.5 (130.75, 169)	0.788
Nibp.Diastolic, median (Q1, Q3)	78 (66, 90)	78 (67, 90)	75.5 (62, 91)	0.144
**Stroke**, ***N*** **(%)**				0.772
No	979 (79)	851 (79)	128 (78)	
Yes	257 (21)	221 (21)	36 (22)	
**Renal disease**, ***N*** **(%)**				0.212
No	1,148 (93)	1,000 (93)	148 (90)	
Yes	88 (7)	72 (7)	16 (10)	
**Diabetes**, ***N*** **(%)**				0.482
No	906 (73)	790 (74)	116 (71)	
Yes	330 (27)	282 (26)	48 (29)	
**Myocardial infarction**, ***N*** **(%)**				0.758
No	1,142 (92)	989 (92)	153 (93)	
Yes	94 (8)	83 (8)	11 (7)	
**Dementia**, ***N*** **(%)**				0.885
No	1,169 (95)	1,013 (94)	156 (95)	
Yes	67 (5)	59 (6)	8 (5)	
**Chronic pulmonary disease**, ***N*** **(%)**				0.493
No	1,136 (92)	988 (92)	148 (90)	
Yes	100 (8)	84 (8)	16 (10)	
**Mild liver disease**, ***N*** **(%)**				0.048
No	1,233 (100)	1,071 (100)	162 (99)	
Yes	3 (0)	1 (0)	2 (1)	
**Hypertension**, ***N*** **(%)**				0.919
No	807 (65)	701 (65)	106 (65)	
Yes	429 (35)	371 (35)	58 (35)	
**Atrial fibrillation**, ***N*** **(%)**				0.15
No	983 (80)	860 (80)	123 (75)	
Yes	253 (20)	212 (20)	41 (25)	
Apsiii, median (Q1, Q3)	35 (26, 50)	33 (25, 46)	51 (37.38, 70)	< 0.001
APACHE II, median (Q1, Q3)	53 (44, 68.62)	51 (43, 65)	69.5 (56, 89)	< 0.001
GCS.Min, median (Q1, Q3)	12 (8, 14)	13 (9, 14)	5.5 (3, 8)	< 0.001
Oasis, median (Q1, Q3)	24 (19, 31)	23 (18, 30)	31 (24.75, 39)	< 0.001

Polys, percentage of polymorphonuclear granulocytes; GCS, Glasgow coma scale; ALT, alanine transaminase; AST, aspartate aminotransferase; ALP, alkaline phosphatase; BUN, blood urea nitrogen; WBC, white blood cells; INR, the international standardization percentage ratio; PT, prothrombin time; SpO_2_, pulse oximetry, APSIII, acute physiology score III. There were significant differences in Physiology Score III; APACHE, Acute physiology and chronic health evaluation II score; OASIS, Oxford acute severity of illness score.

### Selection procedure

The database was randomly divided into two sets: the training set and the validation set in the ratio of 8:2. The recursive feature elimination (RFE) algorithm was used to filter features until the model's AUC value was >0.7.

### Machine learning model development

The present study applied four common machine learning (ML) algorithms to predict the 28-day in-hospital mortality among ICU elderly patients with IS, including Naïve Bayes (NB), eXtreme Gradient Boosting (xgboost), and logistic regression (LR). A validation set of 241 patients who never participated in the model training was used to evaluate all of those ML metrics. To improve the stability of the prediction model, all continuous features are rescaled to a distribution with a mean of 0 and standard deviation of 1, and the scale conversion is performed. After 10-fold cross-validation, the performances of the three prediction models were compared with each other using areas under the curve, specificity, sensitivity, accuracy, positive predictive value (PPV), negative predictive value (NPV), and F1 score. The best model was selected by the area under the receiver operating characteristic curve (AUC) values of the testing set.

### Statistical analysis

Normally distributed continuous data were expressed as the mean with standard errors. To analyze all non-normal homogeneous distributions' continuous characteristics, the Wilcoxon rank sum test was used, which is expressed as the median and interquartile range (IQR). Chi-square analysis or Fisher's exact tests were used to analyze categorical features expressed in frequencies (percentages). Outliers for features will be removed. The imbalance in the distribution of data structures is solved by the SMOTE (synthetic minority oversampling technique) method. The R packages “Nortest” and “CBCgrps” were used for univariate analysis. The RFE function was used for the filtering feature (fivefold cross-validation). Receiver operating characteristic (ROC) mapping and AUC calculation are performed by the “pROC” package, and the interpretability analysis is performed by the “shapviz” package in R (version 4.2.0), data visualization is carried out with the ggplot2 package, and a value of *P* < 0.05 is considered statistically significant.

## Results

### Population demographics

**Figure 1:**
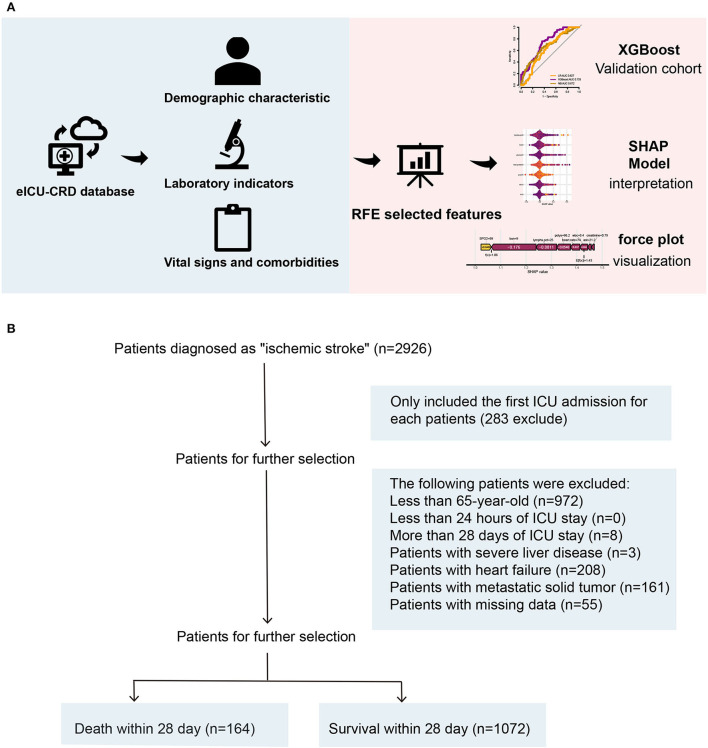
**(A, B)** An overview of the variable selection process for the ML model. ML, machine learning.

A total of 2,435 patients were diagnosed with IS at admission. Finally, a total of 1,518 patients with IS were enrolled in this study according to the inclusion criteria and exclusion criteria (Figure 1), including 636 (51%) women and 600 (49%) men, with a median age of 77 years (IQR, 71–84 years). After 28 days in the hospital, 1,072 patients with IS survived, while 164 died. The differences in characteristics between the survival group and the death group are described in Table 1. A comparison of baseline characteristics after the SMOTE method is shown in the Supplementary Table.

The levels of albumin, blood calcium, bicarbonate, percentage of lymphocytes, percentage of monocytes, percentage of eosinophils, percentage of polymorphonuclear granulocytes (Polys), and Glasgow Coma Scale (GCS) score were much higher in the survival group compared to the death group (*P* < 0.05). However, the levels of bilirubin, alanine transaminase (ALT), aspartate aminotransferase (AST), alkaline phosphatase (ALP), BUN, creatinine, potassium, glucose, WBC, the international standardization percentage ratio (INR), prothrombin time (PT), pulse oximetry (SpO_2_), Acute Physiology Score III (APSIII), Acute Physiology And Chronic Health Evaluation II Score (APACHE II) score, and Oxford Acute Severity of Illness Score (OASIS) were higher in the death group. The death group also had more patients with mild liver disease (*P* < 0.05).

### Feature selection

**Figure 2:**
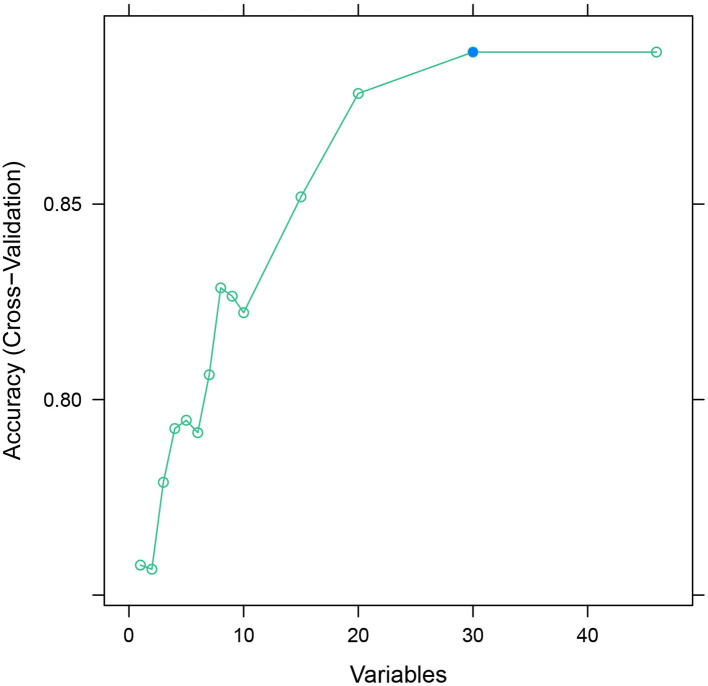
Recursive feature elimination regression analysis.

After feature selection using the recursive feature elimination (RFE) algorithm, eight features remained (Figure 2). Features that included SpO_2_, BUN, percentage of lymphocytes, AST, polymorphonuclear granulocytes (Polys), heart rate, WBC, and creatinine could be used as predictors of the prediction model.

### Model evaluation and comparison

We attempted to use several widely applied machine learning (ML) algorithms (NB, xgboost) for the construction of the prediction models.

**Figure 3:**
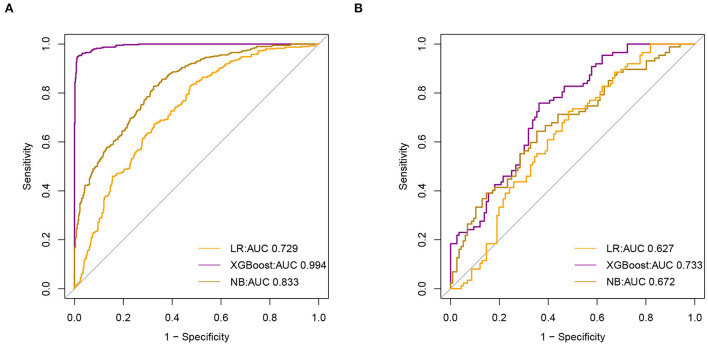
Receiver Operating Characteristic (ROC) curve of model **(A)**. The area under receiver operating characteristic curve (AUC) values of all models in the training set **(B)**. AUC values for all models in the test set.

We used a receiver operating characteristic (ROC) curve, specificity, sensitivity, accuracy, PPV, NPV, and F1 score to evaluate the prediction model in both training and validation data. Before evaluation, optimal cutoffs were determined by maximizing the Youden index (i.e., sensitivity + specificity – 1) by the ROC curve in the validation set. In the validation set, ROC curves revealed that xgboost had the best predictive performances, with an area under the curve of 0.733, better than that of the area under the curves of NB (0.672) and LR (0.627) (Figure 3).

In the training set, the accuracy of the xgboost model (0.994) was higher than the accuracies of the other models. All details about the parameters of the models developed with different algorithms are shown in Table 2.

**Table 2:** Model performance metrics.

**Model**	**F1**	**Accuracy**	**Sensitivity**	**Specificity**	**PPV**	**NPV**
**Training set**					
xgboost	0.963	0.969	0.943	0.989	0.985	0.959
NB	0.695	0.738	0.699	0.767	0.692	0.772
LR	0.555	0.665	0.489	0.796	0.643	0.675
**Validation set**					
xgboost	0.622	0.665	0.644	0.681	0.602	0.718
NB	0.604	0.626	0.667	0.595	0.552	0.704
LR	0.471	0.663	0.442	0.779	0.504	0.732

### Model interpretation

**Figure 4:**
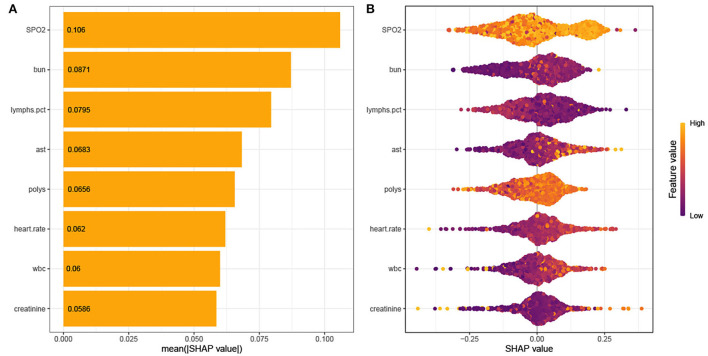
**(A)** SHAP summary plot. **(B)** SHAP bee plot. Interpretation for the eight clinical features contributing to our machine learning (ML) model's prediction for mortality. SpO_2_, oxygen saturation; BUN, blood urea nitrogen; AST, aminotransferase aspartate; Polys, percentage of polymorphonuclear granulocytes; WBC, white blood cells.

As shown in Figure 4, the Tree-Explainer class imported from the SHapley Additive exPlanations (SHAP) package is used to analyze the independent validation set in the xgboost model (27). Figure 4 shows the relationship between the value of the feature and the corresponding SHAP value, which suggests the magnitude of the feature's contribution to the occurrence of the ending event. Following the SHAP summary plot of 28-day death in elderly ICU patients with IS, the associated characteristics with the highest importance score were SpO_2_, BUN, percentage of lymphocytes, AST, Polys, heart rate, WBC, and creatinine.

### ML explainability results for two patients

Using the SHAP force plot, the Shapley value for each feature, which increases (positive value) or decreases (negative value) the prediction from its baseline, was visualized (28). A Shapley value is based on the average of all predictions, and in this case, 53.4% of the held-out validation set.

#### Patient 1

This is an elderly patient who was admitted to the ICU for IS. The patient died on the 28th day. The predicted probability for mortality is high at 72.4%, compared with the baseline of 53.4% (average mortality of the validation set). The features detected by the model for predicting a higher mortality in this patient include Polys, WBC, BUN, AST, and creatinine. In Patient 1, all characteristics, except creatinine, supported the occurrence of the outcome. It was predicted by the ML model that this patient would die, and it truly occurred during admission (true positive).

From a physician's point of view, the ICU observation for this IS patient is reasonable, considering the higher BUN, AST, and creatinine levels in the ICU.

#### Patient 2

**Figure 5:**
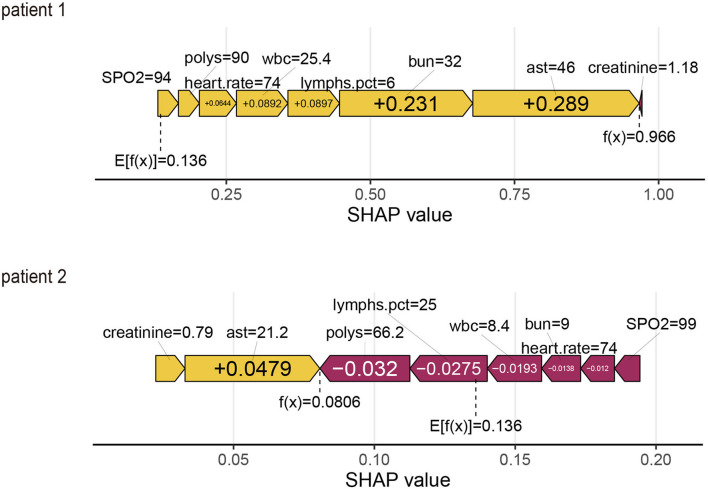
SHapley Additive exPlanations (SHAP) force plot for two patients from the held-out validation set of the machine learning (ML) model.

This is an elderly patient who was admitted to the ICU for IS. The patient survived on the 28th day. The predicted probability for mortality by the model was 52.0% compared with the baseline of 53.4%. The features detected by the model for predicting mortality were all normal. In Patient 2, all features, except creatinine and AST, did not support the occurrence of the outcome. It was predicted by the ML model that this patient would survive, and it occurred during admission (true negative) (Figure 5).

From a physician's perspective, the ICU observation for this IS patient is reasonable, considering the normal values in the ICU.

## Discussion

The novelty of this study is the use of machine learning (ML) to come up with a model that is superior to the traditional LR model. We used this machine learning (ML)-based model that was built on objective indicators to predict the short-term in-hospital mortality risk of elderly people. The model can be used as an automatic warning system to indicate the risk of death of patients. Previous studies proved that the C statistic can improve by 0.12 with the NIHSS score added to the model (29). The C statistic of our model is 0.733, which is higher than those of the previously published models without variables about the disease severity (29, 30). Despite the lack of an NIHSS score, our model was based only on objective indicators that could alert physicians to refine the NIHSS score in time, thus enhancing further the accuracy of the model. With our alerting model, clinical physicians may detect changes in condition in time, which is important for these elderly patients. Moreover, nurses can also provide early warning to indicate the risk of patient death with our alerting model. In addition, for those patients in ICU who have difficulty in body checking or elderly patients with no obvious changes in physical signs, objective indicators would be useful tools to find changes more quickly and timely.

## Main findings

Ischemic stroke is the most common type of stroke, which predominantly affects older adults and has a high short-term mortality rate after admission to the ICU. Therefore, establishing a death prediction model is essential for the risk stratification of these elderly patients with IS.

The present study is the first to use machine learning (ML) models to predict the short-term prognosis of elderly patients with IS admitted to the ICU, which provides a basis for identifying those critically ill older IS patients early. The present study compares the performance of multiple supervised machine learning (ML) algorithms with that of traditional LR methods to explore the best model for predicting short-term death in elderly patients with IS. Each model's prediction performance on the test set resembles that on the training set, indicating that the model has good robustness. In particular, xgboost model based on machine learning shows the best short-term death prediction accuracy compared with those of the traditional LR method and another common machine learning algorithm.

The existing scores for 30-day mortality prediction are PLAN (pre-admission comorbidities, level of consciousness, age, and neurologic deficit) (6) and IScore (5). PLAN was externally validated with an AUC of 0.87 and IScore with an AUC of 0.8. Bonkhoff et al. (16) also reported a predictive model with an AUC of 0.90. Smith et al. (29) established a validation data set prediction performance AUC of 0.72. These models are not designed for elderly patients in the ICU. The present study includes a different population that consists of elderly patients with ischemic stroke in the ICU. Due to a lack of certain variables [PLAN score: stroke subtype, cancer, Canadian Neurological Scale (CNS) score, and symptomatic parameters; IScore: stroke severity, stroke subtype, and cancer; Bonkhoff et al. (16): Situation of living, stroke severity, and symptoms at admission; Smith et al. (29): Mode of arrival, NIHSS score, and smoke], the present study could not externally validate these models and evaluate the applicability for the elderly in the ICU.

In addition, the present study provides a simpler prediction tool that displays clinically useful discrimination of in-hospital risk of mortality for elderly patients with IS in the ICU. Our model is based on readily available variables of the eICU-CRD database, including patient demographics, history, and examination information. The database did not include variables about disease severity such as the Canadian Neurological Scale (CNS) and the National Institutes of Health Stroke Scale (NIHSS). A more widespread use of stroke severity assessments is probably held back by the time needed to complete even a short assessment. NIHSS strongly influences mortality and improvement. When the NIHSS score was added to the model, the C statistic can improve by 0.12 (29). The C statistic of our model is 0.733, which is higher than those of the previously published models without variables about the disease severity (29, 30). This is to say, with the data of NIHSS, our model will improve the C statistic of the xgboost model. While both Wang et al. (7) and our study prove that machine learning was better in the prediction of outcomes in patients with IS than the LR models, our model may present a better performance than PLAN and IScore without NIHSS data.

Some ML models for IS patients (7) have limited clinical applications, and the lack of interpretability of these models is the major barrier. Our results for elderly IS patients in the ICU are more reliable and transparent using the SHAP method, which not only help to get explanations for individual patients but also offer a global explanation for our cohort.

We found elevated oxygen saturation, AST, Polys, heart rate, WBC, and creatinine levels and decreased BUN level, and the percentage of lymphocytes could be used as predictors of the prediction model for 28-day in-hospital mortality in elderly patients with IS admitted to the ICU. Bhatia et al. (31) found impaired consciousness, high total leukocyte count, raised erythrocyte sedimentation rate (ESR), elevated creatinine and ALT, estimated within 24 h of hospitalization, as the most important indicators of 30-day mortality in patients with first-time ischemic stroke. WBC, BUN, and creatinine are associated with the mortality of patients with IS in the ICU (32). Our ML model also emphasizes the importance of WBC, ALT, BUN, and creatinine levels. AST and BUN were reported to be associated with mortality in the ICU (33).

## Limitations

We conceived and developed an observational study that is known to increase the risk of selection bias. Additionally, in the eICU-CRD database, data are collected from electronic health records of 208 hospitals across the country; however, each hospital populates the data differently, which results in significant missing data. Our statistical models did not include variables with more than 30% missing data, which was a major limitation. In addition, the predicting models were built with the US elderly population as a reference; therefore, its application to different ethnic groups and non-elderly people requires further investigation and validation.

## Implications

Predicting mortality helps physicians make better decisions and judgments, as well as coordinate services, communicate with patients, and adjust care plans. Moreover, it provides a reference for assessing stroke mortality and hospital performance.

## Data availability statement

Publicly available datasets were analyzed in this study. This data can be found at: The data analyzed in this study is available at https://eicu-crd.mit.edu/.

## Ethics statement

The eICU-CRD databases were approved by the Massachusetts Institute of Technology (Cambridge, MA) and the Beth Israel Deaconess Medical Center (Boston, MA). The data used for this study are publicly available and de-identified.

## Author contributions

JH, ZL, and XL were responsible for conceiving the study. XD, JD, and HC collected data. WJ, YJ, and TS were responsible for writing the manuscript and for revision of the manuscript. ZL was responsible for designing the study and article processing charge (APC). All authors have approved the submitted version.
