# Supplementary material for: Twenty-eight-day in-hospital mortality prediction for elderly patients with ischemic stroke in the intensive care unit: Interpretable machine learning models
Source: Front Public Health. 2023 Jan 12;10:1086339. doi: 10.3389/fpubh.2022.1086339 (PMC9878123; doi:10.3389/fpubh.2022.1086339)
Supplement: Supplementary file 1 [file Table_1.DOCX]

Supplement Table baseline after SMOTE method

| Variables | Total (n = 931) | Survival (n = 532) | Death (n = 399) | P |
| --- | --- | --- | --- | --- |
| Age, Median (Q1,Q3) | 77.67 (71.56, 83.28) | 77 (70, 83) | 78 (73, 84.13) | 0.004 |
| Gender, n (%) |  |  |  | 0.294 |
| F | 463 (50) | 273 (51) | 190 (48) |  |
| M | 468 (50) | 259 (49) | 209 (52) |  |
| Ethnicity, n (%) |  |  |  | 0.357 |
| Non-white | 239 (26) | 130 (24) | 109 (27) |  |
| White | 692 (74) | 402 (76) | 290 (73) |  |
| BMI, Median (Q1,Q3) | 27.21 (24.1, 31.04) | 27 (23.74, 30.8) | 27.33 (24.52, 31.32) | 0.055 |
| Anion gap, Median (Q1,Q3) | 10 (8, 13) | 10 (8, 13) | 10 (8.12, 12.9) | 0.921 |
| Albumin, Median (Q1,Q3) | 3.6 (3.3, 3.8) | 3.65 (3.3, 3.81) | 3.52 (3.25, 3.7) | < 0.001 |
| Bilirubin, Median (Q1,Q3) | 0.6 (0.5, 0.7) | 0.55 (0.4, 0.7) | 0.6 (0.51, 0.75) | < 0.001 |
| Alt, Median (Q1,Q3) | 22 (18, 27) | 21 (17, 26.5) | 23.4 (19.12, 28.1) | < 0.001 |
| Ast, Median (Q1,Q3) | 22.5 (19, 28) | 21.5 (18, 26) | 24.05 (20.3, 33.38) | < 0.001 |
| Alp, Median (Q1,Q3) | 77.52 (69.68, 92) | 75 (67, 88) | 81.5 (72.65, 96.42) | < 0.001 |
| Bun, Median (Q1,Q3) | 21 (16, 27) | 19 (15, 25) | 23.04 (19.56, 30) | < 0.001 |
| Creatinine, Median (Q1,Q3) | 1.08 (0.86, 1.34) | 1 (0.82, 1.3) | 1.16 (0.93, 1.44) | < 0.001 |
| Sodium, Median (Q1,Q3) | 139 (137, 141) | 139 (137, 141) | 139 (136.76, 141) | 0.834 |
| Calcium, Median (Q1,Q3) | 9 (8.5, 9.4) | 9 (8.6, 9.4) | 8.94 (8.37, 9.35) | 0.012 |
| Chloride, Median (Q1,Q3) | 104 (101, 106.9) | 104 (101, 107) | 104 (101, 106.39) | 0.692 |
| Potassium, Median (Q1,Q3) | 4.04 (3.78, 4.4) | 4 (3.7, 4.3) | 4.2 (3.86, 4.5) | < 0.001 |
| Glucose, Median (Q1,Q3) | 135 (112, 176.56) | 125 (105, 152.25) | 153.46 (122, 202.08) | < 0.001 |
| Bicarbonate, Median (Q1,Q3) | 25 (22.76, 27.02) | 25 (23, 28) | 24.5 (22, 27) | 0.002 |
| Wbc, Median (Q1,Q3) | 9.5 (7.32, 12.27) | 8.6 (7.06, 11.21) | 10.74 (8.18, 13.58) | < 0.001 |
| Hematocrit, Median (Q1,Q3) | 39.12 (35.38, 42.7) | 39.65 (35.58, 42.73) | 38.83 (35.22, 42.48) | 0.436 |
| Hemoglobin, Median (Q1,Q3) | 13 (11.7, 14.3) | 13.2 (11.7, 14.33) | 12.88 (11.63, 14.2) | 0.29 |
| Platelets, Median (Q1,Q3) | 209 (177, 253.86) | 209 (177, 258) | 209.85 (177.81, 249.89) | 0.74 |
| INR, Median (Q1,Q3) | 1.07 (1, 1.17) | 1.04 (1, 1.12) | 1.08 (1, 1.18) | < 0.001 |
| PTT, Median (Q1,Q3) | 28 (26.1, 30.05) | 28 (26.5, 30) | 27.67 (25.82, 30.33) | 0.066 |
| RBC, Median (Q1,Q3) | 4.34 (3.87, 4.74) | 4.38 (3.89, 4.77) | 4.31 (3.86, 4.71) | 0.388 |
| Mcv, Median (Q1,Q3) | 91 (87.88, 94) | 91 (88, 94) | 90.72 (87.7, 94.01) | 0.951 |
| Mchc, Median (Q1,Q3) | 33.3 (32.6, 34) | 33.3 (32.6, 34) | 33.26 (32.61, 34.06) | 0.949 |
| Mch, Median (Q1,Q3) | 30.4 (29.21, 31.63) | 30.48 (29.28, 31.7) | 30.29 (29.21, 31.6) | 0.466 |
| Rdw, Median (Q1,Q3) | 14.09 (13.5, 14.9) | 14 (13.3, 14.9) | 14.19 (13.6, 14.9) | 0.007 |
| Lymphs pct, Median (Q1,Q3) | 17.65 (11.68, 23.62) | 19.5 (13, 26.06) | 15.65 (10.27, 20.17) | < 0.001 |
| Monos pct, Median (Q1,Q3) | 7.6 (6.1, 9) | 7.7 (6.7, 9.11) | 7.3 (5.92, 8.62) | < 0.001 |
| Eos, Median (Q1,Q3) | 1.2 (1, 2) | 1.5 (1, 2.36) | 1.1 (0.91, 1.69) | < 0.001 |
| Polys, Median (Q1,Q3) | 71 (65.65, 77.37) | 69.07 (63, 76.06) | 72.9 (68.39, 78.29) | < 0.001 |
| Total.protein, Median (Q1,Q3) | 6.7 (6.4, 7.1) | 6.7 (6.35, 7.2) | 6.75 (6.45, 7.05) | 0.692 |
| Pt, Median (Q1,Q3) | 13.1 (12.13, 14) | 12.97 (11.84, 14) | 13.26 (12.53, 14.01) | < 0.001 |
| Triglycerides, Median (Q1,Q3) | 105.41 (79, 130) | 105.5 (78, 129.62) | 105 (81.5, 131.25) | 0.484 |
| Total.cholesterol, Median (Q1,Q3) | 156.31 (133.02, 176.25) | 156 (135, 179) | 157 (129, 173.06) | 0.11 |
| Heart.rate, Median (Q1,Q3) | 81 (70.04, 94.56) | 78 (68.75, 90) | 84.05 (73, 98.16) | < 0.001 |
| Respiratory.rate, Median (Q1,Q3) | 18 (16, 20.99) | 18 (16, 21) | 18 (16, 20.65) | 0.665 |
| SPO_2_, Median (Q1,Q3) | 98 (96, 99) | 97 (96, 99) | 98.18 (97, 99.03) | 0.001 |
| nibp.systolic, Median (Q1,Q3) | 150.42 (132.52, 166.22) | 151.5 (132, 167) | 148.75 (132.8, 165.97) | 0.613 |
| nibp.diastolic, Median (Q1,Q3) | 76.5 (65.7, 88.67) | 79 (68, 90) | 73.91 (63, 87) | 0.001 |
| Stroke, n (%) |  |  |  | 0.001 |
| No | 710 (76) | 427 (80) | 283 (71) |  |
| Yes | 221 (24) | 105 (20) | 116 (29) |  |
| Renal Disease, n (%) |  |  |  | 0.032 |
| No | 836 (90) | 488 (92) | 348 (87) |  |
| Yes | 95 (10) | 44 (8) | 51 (13) |  |
| Diabetes, n (%) |  |  |  | < 0.001 |
| No | 641 (69) | 390 (73) | 251 (63) |  |
| Yes | 290 (31) | 142 (27) | 148 (37) |  |
| Myocardial Infarction, n (%) |  |  |  | 0.184 |
| No | 841 (90) | 487 (92) | 354 (89) |  |
| Yes | 90 (10) | 45 (8) | 45 (11) |  |
| Dementia, n (%) |  |  |  | < 0.001 |
| No | 857 (92) | 509 (96) | 348 (87) |  |
| Yes | 74 (8) | 23 (4) | 51 (13) |  |
| Chronic Pulmonary Disease, n (%) |  |  |  | 0.405 |
| No | 845 (91) | 487 (92) | 358 (90) |  |
| Yes | 86 (9) | 45 (8) | 41 (10) |  |
| Mild Liver Disease, n (%) |  |  |  | < 0.001 |
| No | 922 (99) | 532 (100) | 390 (98) |  |
| Yes | 9 (1) | 0 (0) | 9 (2) |  |
| Hypertension, n (%) |  |  |  | < 0.001 |
| No | 574 (62) | 356 (67) | 218 (55) |  |
| Yes | 357 (38) | 176 (33) | 181 (45) |  |
| Atrial Fibrillation, n (%) |  |  |  | < 0.001 |
| No | 702 (75) | 427 (80) | 275 (69) |  |
| Yes | 229 (25) | 105 (20) | 124 (31) |  |
| Apsiii, Median (Q1,Q3) | 41 (29, 60) | 33 (26, 47) | 51.15 (39, 69) | < 0.001 |
| Apache.iva, Median (Q1,Q3) | 60 (46, 77.49) | 51 (43, 66) | 71 (57.74, 86.64) | < 0.001 |
| GCS min, Median (Q1,Q3) | 9.72 (5.62, 13.5) | 13 (10, 14.5) | 5.68 (3.6, 8) | < 0.001 |
| Oasis, Median (Q1,Q3) | 27 (20, 34) | 24 (18, 30) | 30.95 (24.54, 38) | < 0.001 |
